# Phage Therapy for Orthopaedic Infections: The First Three Cases from the United Kingdom

**DOI:** 10.3390/antibiotics14020114

**Published:** 2025-01-22

**Authors:** Daniela I. Munteanu, John Dunn, Gábor Apjok, Bálint Kintses, Johann Griselain, Griet Steurs, Christel Cochez, Sarah Djebara, Maya Merabishvili, Jean-Paul Pirnay, Vida Štilec, Matjaž Peterka, Emily A. Simpson, Samantha Downie, Alasdair MacInnes, Graeme Nicol, Benedict Clift, Joshua D. Jones

**Affiliations:** 1 Department of Medical Microbiology, Ninewells Hospital, Dundee DD1 9SY, UK; 2 Pharmacy Department, Ninewells Hospital, Dundee DD1 9SY, UK; 3 Synthetic and Systems Biology Unit, Institute of Biochemistry, National Laboratory of Biotechnology, HUN-REN Biological Research Centre, Temesvári Krt. 62, 6726 Szeged, Hungary; 4 HCEMM-BRC Translational Microbiology Research Group, Budapesti út 9, 6728 Szeged, Hungary; 5 Laboratory of Molecular and Cellular Technology, Queen Astrid Military Hospital, Bruynstraat 1, 1120 Brussels, Belgium; 6 Center for Infectious Diseases ID4C, Queen Astrid Military Hospital, Bruynstraat 1, 1120 Brussels, Belgium; 7 COBIK, Mirce 21, 5270 Ajdovščina, Slovenia; 8 Trauma and Orthopaedic Department, Ninewells Hospital, Dundee DD1 9SY, UK; 9 Infection Medicine, Edinburgh Medical School: Biomedical Sciences, University of Edinburgh, Chancellor’s Building, 49 Little France Crescent, Edinburgh EH16 4SB, UK

**Keywords:** bacteriophage, bone infection, case series, joint infection, orthopaedic, phage therapy

## Abstract

**Background**: Bacteriophages (phages) are viruses that infect and kill bacteria. The antimicrobial resistance crisis has driven renewed interest in phage therapy, including the use of phages to treat chronic orthopaedic infections. **Methods**: Here, we present the results of the first three orthopaedic patients treated with phage therapy in the United Kingdom. **Results**: The first patient was treated in May 2023 and received phages active against *Staphylococcus aureus*. At nine months follow-up, the patient’s wound remained healed, the C-reactive protein normal and the patient was walking independently. The second patient received phages active against *Klebsiella pneumoniae* and *S. aureus*; the infection remained unresolved. The third patient received phages active against *Staphylococcus epidermidis*; at six months follow-up, the patient was free of infection. Endotoxin was considered at least partially responsible for mild self-limiting adverse effects in two cases. **Conclusions**: These promising results hint at the potential for phage therapy to transform the care of chronic orthopaedic infections.

## 1. Background

Bacteriophages (phages) are naturally occurring viruses that infect and kill bacteria in a generally species-specific manner. Phages were first used to treat bacterial infection in 1919. A lack of knowledge about phages and the mass production of antibiotics saw the demise of phage therapy from mainstream medicine, except in some parts of Eastern Europe [1,2]. Phage therapy has recently experienced a renaissance, driven by the antimicrobial resistance crisis [3]. As there are no licensed phage therapy medicinal products, unlicensed phage therapy is used in cases of special clinical need. A systematic review of 2241 patients with antibiotic refractory infections treated with phage therapy since the year 2000 reported 79% efficacy, 87% bacterial clearance and did not identify any notable adverse effects [4]. Similarly, the available clinical trial data suggest phage therapy is safe, although the methodological shortcomings of previous trials have precluded consistent demonstration of efficacy [5,6,7,8,9,10,11,12]. The United States’ Antibiotic Resistance Leadership Group and Health Improvement Scotland have recommended consideration of phage therapy in antibiotic refractory cases [13,14]. In the United Kingdom (UK), phage therapy has been used clinically in two paediatric respiratory patients and 10 diabetic foot infection patients [15,16].

There is growing interest in phage therapy in orthopaedics, particularly for chronic prosthetic joint infections which are costly to both patients and health authorities. Two systematic reviews of phage therapy for bone and joint infections have reported 71% and 95% efficacy, respectively [17,18]. Phages are typically used as an adjunct to ongoing antibiotic therapy, which offers the potential to exploit the antimicrobial synergy of phages and antibiotics [19]. Phages are particularly attractive to a chronic orthopaedic setting because of their potential activity against biofilm, the bacterial extracellular polysaccharide matrix considered to underlie many chronic infections [20,21]. Here, we present the results of the first three orthopaedic patients treated with phage therapy in the UK.

## 2. Case One

An 84-year-old patient who had undergone a total left hip arthroplasty in 2019 presented three years later with a periprosthetic infection secondary to haematogenous spread from an infected skin biopsy site; a concurrent diagnosis of chronic lymphocytic leukaemia was made. Methicillin-sensitive *Staphylococcus aureus* (MSSA) was initially isolated from blood culture and later from intraoperative samples. Over the next year, the patient underwent a two-stage revision, followed by excision arthroplasty and further surgical debridement. The patient also received prolonged antibiotic therapy, according to sensitivity patterns and the patient’s allergies to penicillin and teicoplanin. However, resolution of infection was not achieved and a sinus and raised inflammatory markers persisted.

Having last been discharged in March 2023, the patient was re-admitted two months later with fever and a computed tomography scan identified recurrent collections within the left hip and ipsilateral sartorius muscle. The patient was considered suitable for unlicensed phage therapy due to the recalcitrance of the infection. Following informed consent, the patient underwent further surgical debridement with intraoperative and postoperative administration of anti-staphylococcal phage therapy. One type of anti-staphylococcal phage (ISP) was used, at a concentration of ≈10^8^ plaque forming units per millilitre (PFU/mL) in 0.9% NaCl and containing endotoxin at 3.7 EU/mL. Phage ISP is a myovirus that was originally discovered by the Eliava Institute (Tbilisi, Georgia) and was manufactured at the Queen Astrid Military Hospital (Brussels, Belgium). The susceptibility of the MSSA isolate to ISP was determined as reported elsewhere [16]. The surgical site including the hip joint and cavity on the anterior thigh was initially rinsed with a 1.26% sodium bicarbonate solution followed by a washout with a suspension of ISP. A short-lived and self-resolving diffuse, non-irritable erythema around the surgical wounds was noted after administration of phages. Drains were placed into each of the two cavities to enable postoperative phage administration. Intraoperative cultures were positive for MSSA and *Pseudomonas aeruginosa*. The patient had been started on intravenous daptomycin before surgical debridement and intravenous ciprofloxacin was added postoperatively when culture results became available to cover for *P. aeruginosa*. Phage therapy was administered via the two drains three times a day, for a total of four days post-surgery followed by removal of the drains. Immediately after the procedure the patient was intermittently febrile (up to 39 °C). A decision to discontinue the local phage administration was made and the fever settled with no more spikes in temperature when phage therapy was resumed. No increases in liver function tests (LFTs) were observed during this time. Cultures from drains and the drain tips were positive only for *P. aeruginosa* and no MSSA was isolated after phage administration.

Two weeks after the initial surgery, the patient had a further washout and debridement for a non-healing wound and rising inflammatory markers. Deep samples from this procedure were again culture-positive for *P. aeruginosa*, but no MSSA was observed; 16S polymerase chain reaction (PCR) for MSSA was also negative. The antibiotic regimen was changed to intravenous ciprofloxacin and meropenem, followed by administration of meropenem alone due to changes in the sensitivity pattern for *P. aeruginosa*. The total duration of antibiotic therapy was six weeks after the last surgery.

A whole-body positron emission tomography-computed tomography was performed six weeks after antibiotics were stopped and the result was consistent with ongoing inflammation, rather than infection. At nine months follow-up, the patient’s wound remained healed, the C-reactive protein was normal and the patient was walking independently. The details of this case have been published elsewhere [22], but the salient features have been summarised here for completeness.

## 3. Case Two

A 71-year-old patient presented with a left periprosthetic hip infection. In 2021, nine years after primary implantation the patient had initial revision surgery for loosening of the acetabular component, followed by a second revision with a cage reconstruction a week later due to a pelvic wall fracture. Within the next two years, the patient had a single stage revision for hip dislocation and further washout and debridement for an ongoing infection. Each time, *Klebsiella pneumoniae* (extended spectrum beta-lactamase) and *Corynebacterium striatum* were isolated. Targeted antibiotic therapy was given each time for 12 weeks and every time shortly after stopping the antibiotics, the patient developed a sinus. In January 2023, phage therapy was discussed with the patient and informed consent obtained. A phage match for the patient’s isolate was not found in existing phage libraries and in March 2023 a ‘Phage Alert’ was issued via Phage Directory [23]. Subsequently, a novel phage highly active against the patient’s *K. pneumoniae* was isolated from a Hungarian wastewater sample and denoted ‘Scrapmetal’ (SCM). Phage SCM was subjected to genotypic and phenotypic characterisation and deemed suitable for clinical use. A purified suspension of 10^9^ PFU/mL phages in 0.9% NaCl containing endotoxin at 3057 EU/mL was produced at the Queen Astrid Military Hospital, Belgium. While phage SCM was being prepared, methicillin-sensitive *Staphylococcus aureus* (MSSA) was isolated in multiple superficial samples from the sinus. The susceptibility of the MSSA isolate to the anti-staphylococcal phage ISP was determined as reported elsewhere [16]. Based on these microbiological findings, the decision was made to administer a cocktail of phages SCM and ISP targeting *K. pneumoniae* and MSSA, respectively. Phages active against *C. striatum* were unavailable and this organism was just targeted with antibiotics to which it was sensitive. As before, a purified suspension of 10^9^ PFU/mL ISP phages in 0.9% NaCl containing endotoxin at 0.5 EU/mL was produced at the Queen Astrid Military Hospital, Belgium. The ISP and SCM phage suspensions were used together at the time of washout and debridement (at 10^8^ and 10^6^ PFU/mL, respectively), following rinsing of the surgical site with a 2.0% sodium bicarbonate solution; at this stage all the metalwork was retained. Phage therapy was used in conjunction with intravenous vancomycin and meropenem. The intraoperative samples were culture-positive for *K. pneumoniae*, MSSA and *C. striatum*. Over the four days following surgery, the patient received seven administrations of postoperative phage therapy via a drain. After four days of postoperative phage therapy, the drain fluid culture was positive for MSSA, with *K. pneumoniae* present only on targeted PCR. The patient experienced fever and nausea in the first two days following the surgery, which subsequently resolved. There was no increase in LFTs or other side-effects. The postoperative wound initially closed but re-opened soon after and continued to leak despite antibiotic treatment and adjustment in the anticoagulant therapy. This led in the end to further washout, debridement and removal of metalwork two months after the initial procedure. Intraoperative cultures taken during debridement were negative after seven days of incubation, but targeted PCR was still positive for both *K. pneumoniae* and MSSA. Due to the complexity of the intervention, some metalwork had to be retained. Consequently, the patient completed a further 12 weeks of antibiotic treatment with vancomycin and meropenem and at discharge the wound was closed and dry.

## 4. Case Three

A 77-year-old patient presented in 2022 with a periprosthetic joint infection of the right knee. The initial implant was performed in 2018 and revised in 2022 for early tibial loosening. Around eight weeks later, the patient was readmitted with a markedly swollen, painful knee. A joint aspirate was carried out which revealed frank pus and debridement, antibiotics and implant retention was undertaken. The intraoperative samples grew *Staphylococcus epidermidis* in three out of five samples. The patient received teicoplanin for 12 weeks, according to the sensitivity profile of the microorganism and patient’s allergy to penicillin. Rifampicin was also started but had to be discontinued after three weeks due to an allergic reaction.

Twelve months later, the patient re-presented with increasing pain in the knee and a sinus; a first-stage revision was performed. Two different strains of *S. epidermidis* were isolated in three out of five intraoperative samples; one of the strains was similar to one identified previously. The patient received a total of six weeks of antibiotics, initially intravenous vancomycin, followed by oral doxycycline. The patient then had a further debridement and insertion of spacer plus further antibiotic therapy for ongoing infection. Due to the intractable nature of the infection, phage therapy was proposed and the patient gave informed consent. Clinical isolates were sent to COBIK (Slovenia), who have previously isolated and characterised phages against *S. epidermidis* [24]. Phage COP-80B was shown by plaque assay to be active against both clinical isolates and purified stocks of 10^9^ PFU/mL COP-80B were prepared containing endotoxin at 0.3 EU/mL. The patient then received anti-staphylococcal phages (10^8^ PFU/mL) at the time of spacer removal and reimplantation. The joint was washed out with the phage suspension following the administration of a 2.0% sodium bicarbonate solution, prior to reimplantation. The patient did not have any side-effects during surgery or in the immediate postoperative phase. Intraoperative samples taken prior to reimplantation were culture-negative. Six months later, the patient was pain-free and the postoperative wound completely healed.

## 5. Discussion

This report presents the results of the first three orthopaedic phage therapy cases from the UK, with two of the three patients achieving a good outcome. This report also represents the first use of phage therapy in the UK against *K. pneumoniae* and *S. epidermidis*. All three cases demonstrate the complexity that bone and joint infections can exhibit. Treatment options were extremely limited in these patients due to multiple allergies and bacteria resistance to multiple antibiotics. In case three, the clinical team decided to proceed with the phage therapy at the time of re-implantation, due to the nature of recalcitrant infection and exclusion of rifampicin as an option, due to allergy, should the infection recur. Although the outcomes in cases one and three were promising, as phage therapy was used in combination with antibiotics and debridement it is impossible to ascribe responsibility for resolution of infection solely to phage therapy, despite laboratory phage sensitivity data.

Clinical trials and observational data both show phage therapy to have a very promising safety profile [4,5]. However, co-administered manufacturing impurities, notably bacterial endotoxin, can cause adverse effects. Local redness at the site of phage administration, as observed here in case one, has been previously observed and attributed to contaminating endotoxin [25]. Endotoxin was also considered to be at least partly responsible for the fever and nausea observed in case two. Elsewhere, raised liver function test (LFTs) results have been inconsistently reported with anti-staphylococcal intravenous and/or intraarticular phage therapy [26,27]. It has been hypothesised that this is unlikely to be directly related to the phages themselves and instead may be driven by innate immune responses associated with biofilm breakdown, although the inconsistency and small number of cases involved mean these results could be anomalous [26,27]. Reassuringly, we did not observe any LFT derangement in our cases.

Successful phage therapy relies on delivering phages throughout the site of infection. Despite laboratory data showing significant bacterial susceptibility to the phages used, we considered the failure of phage therapy in case two to most likely reflect that the phages did not reach some pockets of bacteria. This illustrates how phage therapy must be considered part of a combined approach, alongside rigorous debridement to ensure good delivery of phages and antibiotic therapy.

Although here we elected for local administration of phages, a range of local and/or intravenous approaches to the administration of phage therapy for complex orthopaedic infection have been reported [28]. Resolution of infection has been observed in patients treated with only either intravenous or intraarticular phage therapy. More research is needed on the value of intravenous phage therapy and the levels of tissue penetration achieved.

There is insufficient evidence at this stage to develop robust general treatment protocols and, as an unlicensed medicine, it is important that personalised treatment plans are developed which respond to the specific clinical needs of each case. These plans can be made by local multidisciplinary teams and the governance provided by local NHS unlicenced medicine policies and systems.

Phage therapy has the potential to have a significant impact on the management of complex orthopaedic infections, including prosthetic joint infections—of which around 1% become infected [29]. The cost of managing complex recalcitrant orthopaedic infections is high. For example, it has been estimated that in the five years following primary total hip replacement, patients who develop a prosthetic joint infection and have revision surgery cost approximately £33,000 (over five-fold) more than those who do not [30]. While greater use of unlicensed phage therapy has the potential to make a substantial difference in individual cases, compelling clinical trial data will be required before phage therapy can be integrated into routine care. A clinical trial of phage therapy for orthopaedic infection is ongoing and we await the results with anticipation [31].

## Data Availability

The original contributions presented in this study are included in the article. Further inquiries can be directed to the corresponding author.

## Abbreviations

LFTs, liver function tests; MSSA, methicillin-susceptible Staphylococcus aureus; PCR, polymerase chain reaction; PFU, plaque-forming unit.
